# Short-term effect of household indebtedness and risk of alcohol use disorder among Korean youth: 2017–2020 longitudinal panel study

**DOI:** 10.3389/fpsyt.2023.1189104

**Published:** 2023-07-25

**Authors:** Jinhyun Kim, Il Yun, Hyunkyu Kim, Eun-Cheol Park

**Affiliations:** ^1^Department of Preventive Medicine, Yonsei University College of Medicine, Seoul, Republic of Korea; ^2^Institute of Health Services Research, Yonsei University, Seoul, Republic of Korea; ^3^Department of Psychiatry, Yonsei University College of Medicine, Seoul, Republic of Korea; ^4^Department of Public Health, Graduate School, Yonsei University, Seoul, Republic of Korea

**Keywords:** debt, alcohol misuse, alcohol dependence, CAGE, YP

## Abstract

**Background:**

In Republic of Korea, household debt has increased recently among young adults, especially during the COVID-19 pandemic. Household debt may potentially lead to numerous outcomes including alcohol use disorder (AUD). The aim of this study was to investigate the relationship between a change in indebtedness and the risk of developing AUD.

**Methods:**

A total of 5,091 participants (2,720 men and 2,371 women) were included during a 4-year study period. Indebtedness was divided into four groups: no debt a year ago and at present (group 1), paying off a year’s debt (group 2), newly incurred current debt after a year when there was no debt (group 3), and indebtedness a year ago and at present (group 4). Groups 2, 3, and 4 were also divided into subgroups based on debt characteristics. AUD risk was evaluated by the CAGE scale, and a score of 2 or higher was defined as AUD high risk. Several time-varying socioeconomic and health-related characteristics were adjusted.

**Results:**

Participants who indicated indebtedness at present (groups 3 and 4) were more likely to be AUD high-risk compared to group 1 in both genders (men: adjusted relative risk [aRR] = 1.031, 95% CI [1.014–1.049] in group 3, aRR = 1.028, 95% CI [1.007–1.050] in group 4; women: aRR = 1.039, 95% CI [1.016–1.163] in group 3, aRR = 1.028, 95% CI [1.007–1.050] in group 4). Even paid-off debt affected the risk of AUD among female participants (aRR = 1.018, 95% CI [1.001–1.034] in group 2). Women whose amount of debt increased for 1 year were more likely to be AUD high-risk compared to group 1. Women showed higher aRR than men for increasing CAGE scores by one unit in all debt subgroups.

**Conclusion:**

Our research demonstrated a possible link between indebtedness and a heightened risk of AUD. These results underscore the importance of implementing targeted screening and interventions for AUD, particularly among young women who are facing mounting levels of debt.

## 1. Introduction

Household debt is defined as required principal and interest payments of borrowers in households to lenders at a certain date in the future (1). In Republic of Korea, the ratio of household debt-to-disposable income has increased from 152.9% in 2011 to 206.5% in 2021, which is relatively high among Organization for Economic Co-operation and Development (OECD) countries (2). Among Korean youth (below age 29), the debt-to-asset ratio was 37.1% in 2022, the highest among other age groups (age-average ratio was 16.7% in 2022). There was a significant increase in the ratio during the coronavirus disease pandemic in 2019 (COVID-19), with Korean youth especially vulnerable to economic stress (3).

Household debt can have numerous consequences. It can cause a vicious cycle that involves an increased burden of repaying what is owed and a decrease in investments and corporate productivity (4, 5). With regard to health, a positive association between indebtedness and poor physical and mental health has been found, including depressive symptoms, suicidal risk, and overweight and obesity (6–9). The impact of alcohol use on mortality caused by alcohol-related disorders, with a rate of 9.6 per 100,000 population in 2021, surpasses that of traffic-related fatalities (7.1 per 100,000 population in 2021) and criminal incidents (0.7 per 100,000 population in 2021) (10).

In the context of alcohol consumption and its health-related outcomes, various economic factors have been found to be associated. Notably, higher socioeconomic status (SES) indicators including occupation status, personal or household income, and education level were linked to increased alcohol consumption, and individuals with lower SES experienced higher mortality rates due to alcohol-related causes (11–13). Additionally, the financial crisis in Greece was associated with a decrease in alcohol consumption (14). However, it should be noted that the aforementioned results were derived from cross-sectional data, which limits the ability to establish a temporal relationship between being indebted and alcohol consumption as well as related disorders. Therefore, in our study, we focused on the relationship between the temporal change of indebtedness during 1 year and risk of AUD using the CAGE scale (15). Four types of indebtedness groups were analyzed including no debt a year ago and at present, paying off a year’s debt, newly incurred current debt after a year when there was no debt, and debt a year ago and at present. In addition, the amount of debt and other drinking habits including binge drinking and frequency were considered.

## 2. Materials and methods

### 2.1. Study population and data

The Youth Panel (YP) survey data from 2016 to 2020 by the Republic of Korea Employment Information Service (KEIS) were used in the study. The data period was selected because the dependent variables of this study have been included in the YP survey since 2017, and data from 2016 were applied for a 1-year change in indebtedness. The survey is a nationwide, self-reported, and annual panel study with 15–29 year-old participants. The YP survey has been ongoing since 2007 to collect data on the socioeconomic characteristics of Korean youth to establish youth unemployment policies. Stratified random sampling of households was based on characteristics such as region, age, and gender. All youth participants in one household were included into the panel (an average of 1.49 youth household members per household). The participants in this study were selected from households that were originally recruited through the Occupational Employment Statistics (OES), a nationally recognized official statistic provided by Statistics Republic of Korea. The initial number of participants was 10,206 with 67.7% retention rate in 2020, and 3,516 additional panel participants were included in 2015, which showed a retention rate of 81.6% in 2020. In the initial stage of this analysis (2017), a total of 9,983 participants were included (3,739 participants dropped out), and 4,892 participants who did not answer some survey questions about study variables were excluded. As a result, the data from 5,091 participants (2,720 males and 2,371 women) were analyzed, which consisted of 19,485 observations during the 4-year study period.

## 3. Measures

### 3.1. Household indebtedness

Each year, the participants were repeatedly asked whether they were currently in debt and the amount of debt. Debt contains every sort of required principal and interest payments such as credit card delinquency, bank loans, and debentures. Indebtedness was divided into four groups representing debt status from a year ago to current debt status (written as debt status a year ago—current debt status): No debt–No debt (group 1; no debt a year ago and at present), Debt–No debt (group 2; paying off a year’s debt), No debt–Debt (group 3; newly incurred current debt after a year when there was no debt), and Debt–Debt (group 4; indebtedness a year ago and at present). Group 4 was divided into three subgroups according to the increase or decrease in the amount of debt over the past year: increase in debt amount (group 4–1), the same debt amount (group 4–2), and decrease in debt amount (group 4–3). Furthermore, the amount of debt was divided into two groups based on the median size of the debt in the general population in each survey year and 10-year age group: above median and below median (3). Therefore, groups 2 and 3 were divided into two subgroups and group 4 was divided into four subgroups based on median amount of debt.

### 3.2. CAGE scale for the risk of alcohol use disorder

The CAGE scale is a screening instrument for alcohol abuse that consists of four questions that ask about Cutting down, Annoyance by criticism, Guilty feelings, and Eye-opener (15). As it is a short and easy tool with good reliability and validity (16), more than half of primary physicians have applied the CAGE scale for alcoholism screening (17). The CAGE scale for AUD assessment was administered repeatedly on an annual basis. In this study, Cronbach’s alpha values were found to be 0.59 in 2017, 0.59 in 2018, 0.61 in 2019, and 0.69 in 2020. These results are comparable to previous research examining the application of the CAGE scale in the general population, where Cronbach’s alpha was reported as 0.62 (18). Each question is scored 1 point for “Yes” and zero points for “No.” Although the cutoff score for high risk of AUD is 2, one positive response suggests an alcohol problem according to some studies (19). In our study, a score of 2 or higher was used to classify participants as AUD high risk, and non-drinkers or a score of zero or 1 as AUD low risk. We also assessed binge drinking and frequency of drinking. The standard for binge drinking in Republic of Korea is about 7 glasses per drinking day (equivalent to approximately 60 g of alcohol) for men and 5 glasses per drinking day (equivalent to approximately 40 g of alcohol) for women, occurring on more than one occasion per month over the course of the past year (20). Frequency of drinking was evaluated as how many days participants drink alcohol in a week.

### 3.3. Covariates

Socioeconomic and health-related characteristics were included as covariates. Age was divided into 3 groups: 19–29, 30–39, and 40 and older. Subjective economic status was evaluated by asking “What is your current economic status?” and divided into three groups. Objective economic status was classified based on quintile of household income, which consisted of earned income, financial income, real estate income, and other income. As for the income quintile cutoff for each year, data from Statistics Republic of Korea on the income of the entire population were used (3). Economic activity was divided into two groups based on the question “What did you usually do for the past week?” Paid workers or unpaid family workers with more than 18 h a week were classified as having a job. Area of residence was divided into metropolitan or province (rural). Marital status was divided into two groups: “married” and “single household,” which consisted of single, divorced, separated, and widowed. Education level was divided into two groups: “not more than high school” and “university or higher.” Self-report health status was evaluated by asking participants, “How do you think your health status is?” and divided them into three groups. Smoking status was divided into “non-smoker” and “ever-smoker.” Sleep duration was divided into two groups based on the recommended duration for adults, which is 7 h (21). Physical activity was classified into “Yes or “No by asking “How many hours do you exercise a week on average?”

### 3.4. Statistical analysis

The statistics for the socioeconomic and health-related variables were conducted using the chi-square test. Generalized estimating equation (GEE) regression was applied and the temporal variable was survey year to investigate repeated measures variables. A Poisson regression model with a log link function and autoregressive working correlation matrix, which was the minimum QIC statistic, was used. Multivariate GEE regression was adjusted by covariates. Results were written as adjusted risk ratios (aRR) with 95% confidence intervals (CI) to represent the effect of 1-year temporal alteration for indebtedness on a current CAGE score of 2 or more. To investigate the detailed effect based on amount of debt and covariates, subgroup analyses were conducted. In addition, relative risk of a CAGE score of 1 compared to a CAGE score of zero was analyzed. Weighted variables for dropout given by YP survey were included in the weighted regression analysis. Because all variance inflation factors were less than 1.4, there was no evidence of multicollinearity. SAS version 9.4 software (SAS Institute, Cary, NC, USA) was used for data analysis and the *P*-value of 0.05 was used for the level of statistical significance.

## 4. Results

General characteristics of the participants at the initial period of our study are shown in Table 1. Among men (*n* = 2,720, 53.4%), 2.8% were AUD high-risk and 1.8% of women (*n* = 2,371, 46.6%) were AUD high-risk. Based on 1-year indebtedness change, the percentage of AUD high-risk was 1.8% in group 1, 3.8% in group 2, 6.4% in group 3, and 5.5% in group 4 among men. The percentage of AUD high-risk among women was 1.1, 4.0, 4.2, and 7.5%, respectively.

**TABLE 1:** General characteristics of participants as of the initial period of our study stratified by gender.

Variables	Male (*N* = 2720, 53.4%)					Female (*N* = 2371, 46.6%)				
	AUD low-risk		AUD high-risk		*p*-value	AUD low-risk		AUD high-risk		*p*-value
	*N*	(%)	*N*	(%)		*N*	(%)	*N*	(%)	
**Total**	2644	(97.2)	76	(2.8)		2329	(98.2)	42	(1.8)	
**1-year indebtedness change**					**<0.0001**					**<0.0001**
No debt–No debt (group 1)	1975	(98.2)	37	(1.8)		1964	(98.9)	21	(1.1)	
Debt–No debt (group 2)	125	(96.2)	5	(3.8)		72	(96.0)	3	(4.0)	
No debt–Debt (group 3)	219	(93.6)	15	(6.4)		158	(95.8)	7	(4.2)	
Debt–Debt (group 4)	325	(94.5)	19	(5.5)		135	(92.2)	11	(7.5)	
**Age**					0.1411					0.6333
19∼29	707	(98.3)	12	(1.7)		936	(97.5)	24	(2.2)	
30∼39	1664	(96.9)	54	(3.1)		1216	(98.7)	16	(1.3)	
More than 40	273	(96.5)	10	(3.5)		177	(98.9)	2	(1.1)	
**Subjective economic status**					0.0530					**<0.0001**
Low	397	(95.9)	17	(4.1)		291	(94.8)	16	(5.2)	
Middle	1811	(97.5)	46	(2.2)		1555	(98.7)	21	(1.3)	
High	436	(97.1)	13	(2.9)		483	(99.0)	5	(1.0)	
**Objective economic status**					0.3352					0.7683
Lowest quintile	415	(96.5)	15	(3.5)		388	(97.7)	9	(2.3)	
Second quintile	507	(98.6)	7	(1.4)		278	(97.9)	6	(2.1)	
Middle quintile	687	(97.9)	15	(2.1)		412	(97.6)	10	(2.4)	
Fourth quintile	685	(96.6)	24	(3.4)		860	(98.7)	11	(1.3)	
Top quintile	350	(95.9)	15	(4.1)		391	(98.5)	6	(1.5)	
**Economic activity**					0.2327					0.3742
Absence	55	(94.8)	3	(5.2)		118	(97.5)	3	(2.2)	
Existence	2589	(97.3)	73	(2.7)		2211	(98.3)	39	(1.7)	
**Area of residence**					0.3616					**0.0004**
Metropolitan	2095	(96.9)	66	(3.1)		1908	(98.0)	38	(2.0)	
Province (rural)	549	(98.2)	10	(1.8)		421	(99.1)	4	(0.9)	
**Marital status**					0.0901					0.2043
Single household	1538	(97.9)	33	(2.1)		1523	(97.9)	32	(2.1)	
Married	1106	(96.3)	43	(3.7)		806	(98.8)	10	(1.2)	
**Education level**					0.8246					0.3953
Not more than high school	602	(97.7)	14	(2.3)		359	(97.0)	11	(3.0)	
University or higher	2042	(97.1)	62	(2.9)		1970	(98.5)	31	(1.5)	
**Self-report health status**					**0.0050**					0.4805
High	1885	(98.0)	38	(2.0)		1430	(98.6)	20	(1.4)	
Middle	525	(95.5)	25	(4.5)		588	(98.2)	11	(1.8)	
Low	234	(94.7)	13	(5.3)		311	(96.6)	11	(3.4)	
**Smoking status**					0.6675					**0.0007**
Non-smoker	1891	(97.2)	55	(2.8)		2296	(98.4)	38	(1.6)	
Ever-smoker	753	(97.3)	21	(2.7)		33	(89.2)	4	(10.8)	
**Sleep duration**					**0.0149**					0.0170
More than 7-h	1909	(97.7)	45	(2.3)		1748	(98.6)	25	(1.4)	
Less than 7-h	735	(96.0)	31	(4.0)		581	(97.2)	17	(2.8)	
**Physical activity**					0.8848					0.2368
No	1111	(97.0)	34	(3.0)		1371	(98.8)	17	(1.2)	
Yes	1533	(97.3)	42	(2.7)		958	(97.5)	25	(2.2)	

Bold values mean *p* < 0.05 which is statistically significant.

The results of GEE regression adjusting for the covariates, which represents the relationship between temporal change of indebtedness and AUD high-risk, are shown in Table 2. Compared to men who had no debt a year ago and at present (group 1), men who had debt currently (groups 3 and 4) were more likely to be AUD high-risk (aRR = 1.031, 95% CI [1.014–1.049] in group 3, aRR = 1.028, 95% CI [1.007–1.050] in group 4). Women who had debt a year ago or at present (groups 2, 3, and 4) were more likely to be AUD high-risk compared to women who had no debt a year ago and at present (aRR = 1.018, 95% CI [1.001–1.034] in group 2, aRR = 1.039, 95% CI [1.016–1.163] in group 3, aRR = 1.028, 95% CI [1.007–1.050] in group 4).

**TABLE 2:** The relationship between temporal change of indebtedness during 1-year and AUD high-risk.

Variables	Alcohol use disorder high-risk							
	Male (*N* = 2720, 53.4%)				Female (*N* = 2371, 46.6%)			
	aRR	95% CI			aRR	95% CI		
**1-year indebtedness change**								
No debt–No debt (group 1)	1.000				1.000			
Debt–No debt (group 2)	1.014	0.998	–	1.030	1.018*	1.001	–	1.034
No debt–Debt (group 3)	1.031*	1.014	–	1.049	1.039*	1.016	–	1.063
Debt–Debt (group 4)	1.028*	1.009	–	1.047	1.028*	1.007	–	1.050
**Age**								
19∼29	1.000				1.000			
30∼39	1.006	0.996	–	1.017	1.000	0.991	–	1.009
More than 40	1.010	0.992	–	1.029	0.988	0.976	–	1.000
**Subjective economic status**								
Low	1.000				1.000			
Middle	0.985*	0.973	–	0.997	0.978*	0.963	–	0.994
High	0.991	0.974	–	1.007	0.979*	0.963	–	0.995
**Objective economic status**								
Lowest quintile	1.000				1.000			
Second quintile	0.990	0.977	–	1.004	1.004	0.989	–	1.018
Middle quintile	0.999	0.985	–	1.014	1.002	0.990	–	1.014
Fourth quintile	1.003	0.990	–	1.016	1.007	0.996	–	1.017
Top quintile	1.016	0.999	–	1.034	1.005	0.993	–	1.018
**Economic activity**								
Absence	1.000				1.000			
Existence	0.979	0.939	–	1.022	0.999	0.981	–	1.017
**Area of residence**								
Metropolitan	1.000				1.000			
Province (rural)	0.993	0.981	–	1.004	0.997	0.988	–	1.006
**Marital status**								
Single household	1.000				1.000			
Married	0.996	0.985	–	1.008	0.992	0.984	–	1.001
**Education level**								
Not more than high school	1.000				1.000			
University or higher	0.999	0.986	–	1.012	1.000	0.990	–	1.011
**Self-report health status**								
High	1.000				1.000			
Middle	1.029*	1.016	–	1.041	1.019*	1.009	–	1.029
Low	1.030*	1.010	–	1.051	1.029*	1.008	–	1.050
**Smoking status**								
Non-smoker	1.000				1.000			
Ever-smoker	1.010	0.998	–	1.023	1.063	0.987	–	1.145
**Sleep duration**								
More than 7-h	1.000				1.000			
Less than 7-h	1.018*	1.004	–	1.032	1.000	0.991	–	1.011
**Physical activity**								
No	1.000				1.000			
Yes	0.998	0.988	–	1.008	1.006	0.998	–	1.015

*Statistically significant. aRR, adjusted relative risk; CI, confidence interval.

**FIGURE 1:**
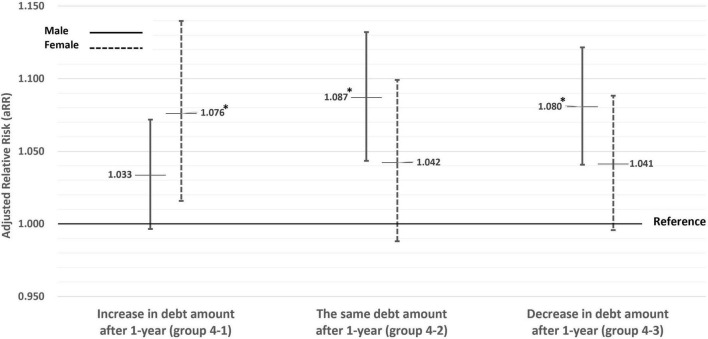
Subgroup analyses based on the increase or decrease in the amount of debt over the past year (in group 4, indebtedness a year ago and at present). Adjusted by age, subjective economic status, objective economic status, economic activity, area of residence, marital status, education level, self-report health status, smoking status, sleep duration, and physical activity. *Statistically significant.

Among group 4, men whose amount of debt was decreased (group 4–1) or the same (group 4–2) for 1 year or women whose amount of debt was increased for 1 year (group 4–3) were more likely to be AUD high-risk compared to group 1 (Figure 1). Above median indebtedness a year ago and at present were more likely to be AUD high-risk compared to group 1 in both genders (aRR = 1.035, 95% CI [1.003–1.068] among men, aRR = 1.038, 95% CI [1.002–1.077] among women) (Supplementary Figure 1). Among both genders in the subgroups in which the relative risk of being AUD high-risk for current indebtedness (groups 3 and 4) compared to group 1, engaging in economic activity, a higher level of education, being a non-smoker, and sleeping at least 7 h contributed to being AUD high risk (Supplementary Table 1).

**FIGURE 2:**
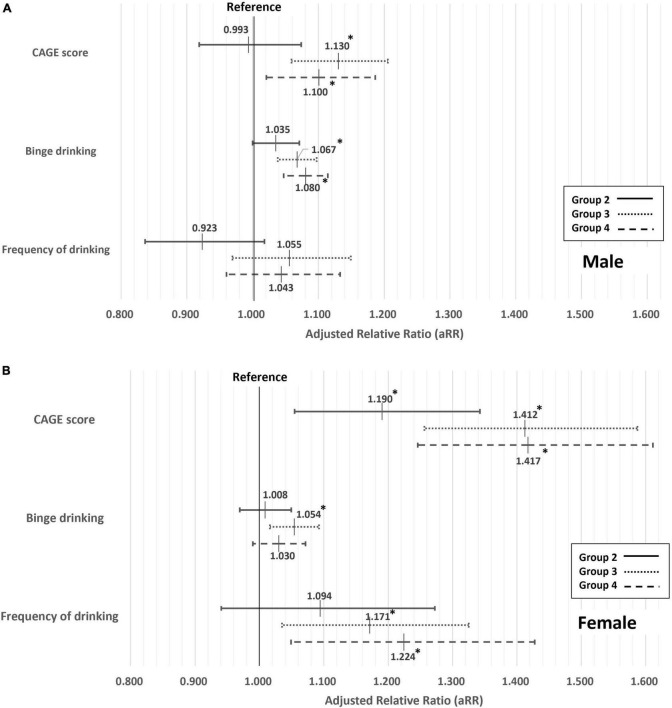
Association between the temporal change of indebtedness and CAGE score divided by 1 score-unit, binge drinking, and frequency of drinking. Adjusted by age, subjective economic status, objective economic status, economic activity, area of residence, marital status, education level, self-report health status, smoking status, sleep duration, and physical activity. *Statistically significant. Among males **(A)** and among females **(B)**.

The results of the multivariate Poisson regression between the temporal change of indebtedness and CAGE score divided by 1 score-unit, binge drinking, and frequency of drinking are shown in Figure 2. Compared to group 1, group 3 was 1.130 times and group 4 was 1.100 times more likely to have an increase in CAGE score by 1 unit among men, and groups 2, 3, and 4 were, respectively, 1.190 times, 1.412 times, and 1.417 times more likely to have an increase in CAGE score by 1 unit among women. Compared to group 1, groups 3 and 4 among men and group 3 among women were more likely to experience binge drinking. Only among women, groups 3 and 4 were more likely to drink 1 day more per week compared to group 1.

## 5. Discussion

This study aimed to determine the effect of 1-year indebtedness change on risk of AUD among Korean youth using data from 2017 to 2020. The 1-year indebtedness change variable consisted of 4 options. In both genders, current indebtedness was associated with AUD high-risk. Additionally, other drinking habits were considered and the effect of the amount of debt was investigated in a subgroup analysis.

There is some previous research on the relationship between indebtedness and alcohol use disorder. According to a cross-sectional study in England, Scotland, and Wales, there was a positive relationship between the amount of dept and Alcohol Use Disorder Identification Test score (22). In an OECD study, short-term (<1 year), middle-term (1–5 years), and long-term (>5 years) debt was associated with more liters of alcohol consumption (23). In contrast, among US adults, three groups based on the proportion of debt to income (zero, up to half, and more than half) showed no significant difference for number of alcohol drinks per week (24). In addition, there was no association between indebtedness and alcohol consumption that was evaluated by asking “Have you drunk more than 3 times a week during the past month” among Chinese residents (25).

In our study, being indebted at present (groups 3 and 4) was associated with higher CAGE scores (2 or more) regardless of socioeconomic status in both genders. Women showed higher aRRs than men for increasing CAGE scores by 1 unit. In addition, even paid off debt (group 2) affected the risk of AUD. In addition, even paid off debt (group 2) affected the risk of AUD among women. According to previous research, it has been consistently observed that women exhibit greater vulnerability to stress arising from indebtedness, regardless of their income level or the amount of debt, compared to men (26, 27). In light of this finding, it is plausible to suggest that even paid-off debt may potentially contribute to the development of AUD among women, as the phenotype of psychological stress associated with indebtedness. Meanwhile, men and women showed different patterns of alcohol use, which were affected by indebtedness. Women whose debt had increased after 1 year were more at risk for AUD. Furthermore, with regard to indebtedness at present regardless of a year ago debt status, women were more likely to drink 1 day more per week compared to the no debt group. In contrast, current indebtedness was not associated with frequency of drinking in men, but they were more likely to binge drink. A similar trend for binge drinking was shown among women with new indebtedness compared to a year ago.

There are some well-known risk factors for AUD, such as genetic predisposition, other mental disorders, personality, perceived stress level, poor family support, and permissive cultural attitudes toward alcohol (28–31). Indebtedness was also associated with these risk factors, including perceived stress level and other mental disorders (32, 33). It could be possible that indebtedness affects AUD risk through several common factors, but this is not clear. Although there would be complex mechanisms among indebtedness, high-risk AUD, and related factors, it is expected that negative consequences due to AUD are likely because of increasing repayment burdens with increasing base interest rates since August 2021 (34).

Meanwhile, AUD is a major psychiatric problem because of its high past-year prevalence (as of 2021, 3.4% of men and 1.8% of women) and relatively higher prevalence among Korean youth than other age groups (35). It is important to highlight that the average past-year prevalence of AUD among women in 27 countries, excluding Republic of Korea, was reported as 0.9%, which is only half of the prevalence observed in Republic of Korea among women (36). This indicates that AUD among women in Republic of Korea presents an even more pronounced social and medical issue that demands attention. In addition, AUD is associated with suicide (37), which is the most common cause of death among Korean youth (as of 2021, 56.8% for people in their 20 s and 40.6% for people in their 30 s) (38). Alcohol dependence in youth could affect poor late-stage brain development, higher lifetime prevalence of alcohol use disorder as well as other physical diseases such as liver disease (39–42). Therefore, to effectively prevent alcohol use disorder (AUD) and mitigate its consequences, it is imperative to implement appropriate population-level interventions. These interventions should encompass regulations related to alcohol availability, such as taxation policies, restrictions on alcohol sales based on hours or locations, and limitations on alcohol advertisements targeting youth. These measures have consistently demonstrated their effectiveness in previous research (43). Additionally, conducting comprehensive education campaigns and integrating an AUD screening tool into national check-ups could serve as valuable additional options. Further research could be conducted to explore the prevalence of AUD among youth based on the reasons of indebtedness using more rigorous assessment methods. Additionally, the effectiveness of interventions aimed at reducing the prevalence of AUD, particularly among youth could be researched.

This study has several limitations. First, the CAGE scale, as provided in the survey, demonstrated relatively low validity (44) for AUD among the general population and exhibited a low Cronbach’s alpha in this study. Moreover, the absence of alternative evaluation tools for AUD further compounds this limitation. Also, debt status was evaluated by self-report; therefore, low data accuracy is inevitable. Second, other mental disorders or causes of indebtedness could be potential covariates, but they were not included in this investigation because such data was not collected in the YP survey. Lastly, these results are based on a large sample of Korean youth; they cannot be generalized to other age groups or countries and ethnicities with different cultures.

In conclusion, the short-term effect of household indebtedness and risk of AUD among Korean youth was shown in the study. Indebtedness at present in both genders was associated with AUD high-risk, and even paid-off debt could affect the risk of AUD among women. Appropriate screening and interventions for AUD among Korean youth in debt need to be considered.

## Data availability statement

The original contributions presented in this study are included in the article/Supplementary material, further inquiries can be directed to the corresponding author.

## Ethics statement

Ethical review and approval was not required for the study on human participants in accordance with the local legislation and institutional requirements. Written informed consent for participation was not required for this study in accordance with the national legislation and the institutional requirements.

## Author contributions

E-CP conceived, designed, directed the study, critically reviewed the manuscript, had primary responsibility for final content, and responsible for the revision of the manuscript. JK conceptualized the study, conducted statistical analysis, interpreted the data, wrote the initial draft of the manuscript, and revised the manuscript. IY and HK conducted the statistical analyses of the data and critically reviewed the manuscript. All authors participated sufficiently in the study and read and approved the final manuscript.
