# Supplementary material for: Short-term effect of household indebtedness and risk of alcohol use disorder among Korean youth: 2017–2020 longitudinal panel study
Source: Front Psychiatry. 2023 Jul 25;14:1189104. doi: 10.3389/fpsyt.2023.1189104 (PMC10407130; doi:10.3389/fpsyt.2023.1189104)
Supplement: Supplementary file 1 [file Data_Sheet_1.docx]

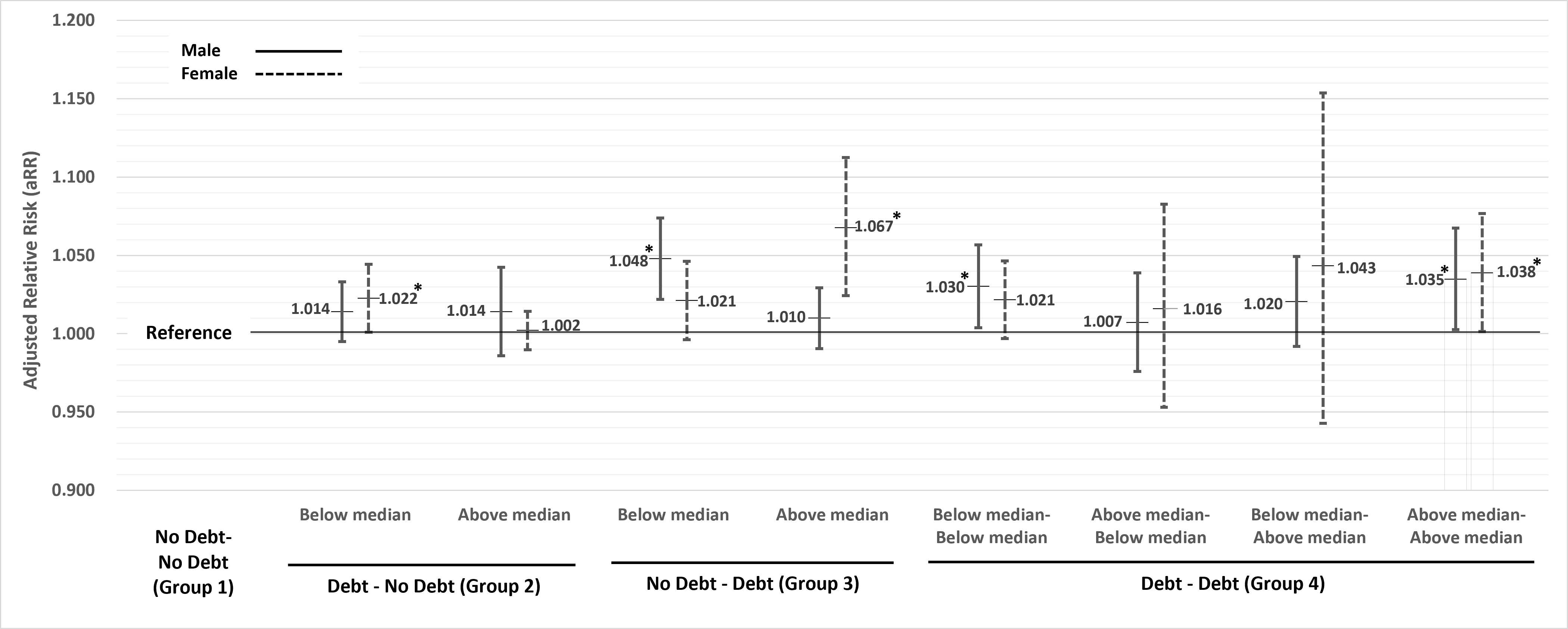
**Supplementary Figure 1** Subgroup analyses based on the median debt amount for a general population of the same age group

* Statistically significant

**Supplementary Table 1** Subgroup analyses based on socioeconomic and health-related factors

| **Variables** | | **Alcohol Use Disorder High-risk** | | | | | | | | | | | | | | |
| --- | --- | --- | --- | --- | --- | --- | --- | --- | --- | --- | --- | --- | --- | --- | --- | --- |
|  |  | **Group 1** | **Group 2** | | | | **Group 3** | | | | **Group 4** | | | | |  |
|  |  | **Adjusted RR** | **Adjusted RR** | **95% CI** | | | **Adjusted RR** | **95% CI** | | | **Adjusted RR** | **95% CI** | | | |  |
|  | **Male (N=2720, 53.4%)** | | | | | | | | | | | | | |  | |
| **Age** | |  |  |  |  |  |  |  |  |  |  |  |  |  | |  |
|  | 19~29 | 1.000 | 0.995 | 0.969 | - | 1.021 | 1.019 | 0.987 | - | 1.053 | 1.035 | 0.990 | - | 1.082 | |  |
|  | 30~39 | 1.000 | 1.018 | 0.999 | - | 1.038 | 1.038 | 1.016 | - | 1.061 | 1.027 | 1.004 | - | 1.050 | |  |
|  | More than 40 | 1.000 | 1.000 | 0.953 | - | 1.049 | 1.009 | 0.967 | - | 1.052 | 1.025 | 0.983 | - | 1.070 | |  |
| **Subjective economic status** | |  |  |  |  |  |  |  |  |  |  |  |  |  | |  |
|  | Low | 1.000 | 1.015 | 0.972 | - | 1.059 | 1.030 | 0.987 | - | 1.075 | 1.017 | 0.983 | - | 1.053 | |  |
|  | Middle | 1.000 | 1.019 | 0.998 | - | 1.039 | 1.024 | 1.007 | - | 1.042 | 1.022 | 1.001 | - | 1.044 | |  |
|  | High | 1.000 | 0.995 | 0.967 | - | 1.025 | 1.056 | 0.991 | - | 1.125 | 1.064 | 0.992 | - | 1.140 | |  |
| **Objective economic status** | |  |  |  |  |  |  |  |  |  |  |  |  |  | |  |
|  | Lowest quintile | 1.000 | 0.985 | 0.955 | - | 1.015 | 1.014 | 0.978 | - | 1.052 | 1.015 | 0.971 | - | 1.061 | |  |
|  | Second quintile | 1.000 | 1.040 | 0.999 | - | 1.082 | 1.025 | 0.999 | - | 1.053 | 1.025 | 0.998 | - | 1.053 | |  |
|  | Middle quintile | 1.000 | 1.014 | 0.989 | - | 1.039 | 1.052 | 1.012 | - | 1.093 | 1.018 | 0.987 | - | 1.049 | |  |
|  | Fourth quintile | 1.000 | 1.025 | 0.989 | - | 1.063 | 1.015 | 0.987 | - | 1.044 | 1.021 | 0.987 | - | 1.055 | |  |
|  | Top quintile | 1.000 | 0.970 | 0.951 | - | 0.988 | 1.042 | 0.980 | - | 1.109 | 1.067 | 1.000 | - | 1.137 | |  |
| **Economic activity** | |  |  |  |  |  |  |  |  |  |  |  |  |  | |  |
|  | Absence | 1.000 | 1.000 | 0.999 | - | 1.002 | 0.975 | 0.883 | - | 1.078 | 1.040 | 0.951 | - | 1.136 | |  |
|  | Existence | 1.000 | 1.014 | 0.998 | - | 1.030 | 1.033 | 1.015 | - | 1.051 | 1.025 | 1.007 | - | 1.045 | |  |
| **Area of residence** | |  |  |  |  |  |  |  |  |  |  |  |  |  | |  |
|  | Metropolitan | 1.000 | 1.020 | 1.000 | - | 1.041 | 1.032 | 1.012 | - | 1.053 | 1.028 | 1.005 | - | 1.051 | |  |
|  | Province(rural) | 1.000 | 1.000 | 0.978 | - | 1.023 | 1.030 | 0.994 | - | 1.067 | 1.026 | 0.991 | - | 1.062 | |  |
| **Marital status** | |  |  |  |  |  |  |  |  |  |  |  |  |  | |  |
|  | Not married | 1.000 | 1.006 | 0.983 | - | 1.029 | 1.036 | 1.005 | - | 1.068 | 1.028 | 0.992 | - | 1.066 | |  |
|  | Married | 1.000 | 1.020 | 0.998 | - | 1.043 | 1.027 | 1.008 | - | 1.047 | 1.025 | 1.004 | - | 1.046 | |  |
| **Education level** | |  |  |  |  |  |  |  |  |  |  |  |  |  | |  |
|  | Not more than high school | 1.000 | 0.979 | 0.957 | - | 1.002 | 1.054 | 1.008 | - | 1.103 | 1.041 | 0.995 | - | 1.090 | |  |
|  | University or higher | 1.000 | 1.024 | 1.004 | - | 1.044 | 1.023 | 1.006 | - | 1.041 | 1.024 | 1.004 | - | 1.045 | |  |
| **Self-report health status** | |  |  |  |  |  |  |  |  |  |  |  |  |  | |  |
|  | High | 1.000 | 1.015 | 0.998 | - | 1.032 | 1.019 | 1.002 | - | 1.037 | 1.028 | 1.008 | - | 1.049 | |  |
|  | Middle | 1.000 | 0.005 | 0.962 | - | 1.050 | 1.061 | 1.018 | - | 1.106 | 1.029 | 0.994 | - | 1.066 | |  |
|  | Low | 1.000 | 0.952 | 0.897 | - | 1.009 | 1.009 | 0.892 | - | 1.142 | 0.992 | 0.894 | - | 1.101 | |  |
| **Smoking status** | |  |  |  |  |  |  |  |  |  |  |  |  |  | |  |
|  | Non-smoker | 1.000 | 1.017 | 0.997 | - | 1.036 | 1.022 | 1.005 | - | 1.040 | 1.033 | 1.011 | - | 1.056 | |  |
|  | Ever-smoker | 1.000 | 1.001 | 0.973 | - | 1.031 | 1.055 | 1.011 | - | 1.100 | 1.010 | 0.974 | - | 1.047 | |  |
| **Sleep duration** | |  |  |  |  |  |  |  |  |  |  |  |  |  | |  |
|  | More than 7-hour | 1.000 | 1.009 | 0.992 | - | 1.026 | 1.028 | 1.008 | - | 1.048 | 1.024 | 1.002 | - | 1.047 | |  |
|  | Less than 7-hour | 1.000 | 1.020 | 0.984 | - | 1.058 | 1.037 | 1.001 | - | 1.074 | 1.031 | 0.995 | - | 1.068 | |  |
| **Physical activity** | |  |  |  |  |  |  |  |  |  |  |  |  |  | |  |
|  | No | 1.000 | 1.045 | 1.013 | - | 1.079 | 1.055 | 1.022 | - | 1.088 | 1.043 | 1.006 | - | 1.082 | |  |
|  | Yes | 1.000 | 0.995 | 0.977 | - | 1.012 | 1.017 | 0.997 | - | 1.038 | 1.017 | 0.994 | - | 1.041 | |  |
|  | **Female (N=2371, 46.6%)** | | | | | | | | | | | | | |  | |
| **Age** | |  |  |  |  |  |  |  |  |  |  |  |  |  | |  |
|  | 19~29 | 1.000 | 1.020 | 0.991 | - | 1.050 | 1.029 | 0.993 | - | 1.066 | 1.050 | 1.003 | - | 1.100 | |  |
|  | 30~39 | 1.000 | 1.014 | 0.994 | - | 1.035 | 1.042 | 1.010 | - | 1.076 | 1.021 | 0.997 | - | 1.046 | |  |
|  | More than 40 | 1.000 | 1.025 | 0.975 | - | 1.078 | 1.024 | 0.986 | - | 1.065 | 1.022 | 0.977 | - | 1.069 | |  |
| **Subjective economic status** | |  |  |  |  |  |  |  |  |  |  |  |  |  | |  |
|  | Low | 1.000 | 1.023 | 0.964 | - | 1.087 | 1.045 | 0.999 | - | 1.094 | 1.047 | 0.989 | - | 1.109 | |  |
|  | Middle | 1.000 | 1.021 | 1.002 | - | 1.041 | 1.033 | 1.004 | - | 1.064 | 1.021 | 1.000 | - | 1.043 | |  |
|  | High | 1.000 | 0.992 | 0.983 | - | 1.000 | 1.055 | 0.993 | - | 1.120 | 1.001 | 0.978 | - | 1.024 | |  |
| **Objective economic status** | |  |  |  |  |  |  |  |  |  |  |  |  |  | |  |
|  | Lowest quintile | 1.000 | 1.008 | 0.968 | - | 1.051 | 1.062 | 0.980 | - | 1.151 | 1.029 | 0.986 | - | 1.073 | |  |
|  | Second quintile | 1.000 | 0.995 | 0.952 | - | 1.039 | 1.031 | 0.981 | - | 1.083 | 1.025 | 0.974 | - | 1.079 | |  |
|  | Middle quintile | 1.000 | 1.009 | 0.989 | - | 1.029 | 1.050 | 0.992 | - | 1.111 | 1.095 | 1.020 | - | 1.177 | |  |
|  | Fourth quintile | 1.000 | 1.023 | 0.997 | - | 1.051 | 1.036 | 1.000 | - | 1.074 | 1.012 | 0.987 | - | 1.038 | |  |
|  | Top quintile | 1.000 | 1.027 | 0.976 | - | 1.080 | 1.028 | 0.978 | - | 1.080 | 1.008 | 0.981 | - | 1.036 | |  |
| **Economic activity** | |  |  |  |  |  |  |  |  |  |  |  |  |  | |  |
|  | Absence | 1.000 | 0.980 | 0.956 | - | 1.004 | 1.006 | 0.933 | - | 1.084 | 1.035 | 0.964 | - | 1.111 | |  |
|  | Existence | 1.000 | 1.020 | 1.002 | - | 1.037 | 1.040 | 1.016 | - | 1.065 | 1.028 | 1.006 | - | 1.051 | |  |
| **Area of residence** | |  |  |  |  |  |  |  |  |  |  |  |  |  | |  |
|  | Metropolitan | 1.000 | 1.016 | 0.995 | - | 1.036 | 1.037 | 1.013 | - | 1.061 | 1.022 | 0.999 | - | 1.046 | |  |
|  | Province(rural) | 1.000 | 1.020 | 0.997 | - | 1.044 | 1.047 | 0.987 | - | 1.110 | 1.047 | 0.997 | - | 1.099 | |  |
| **Marital status** | |  |  |  |  |  |  |  |  |  |  |  |  |  | |  |
|  | Not married | 1.000 | 1.020 | 0.992 | - | 1.048 | 1.044 | 1.006 | - | 1.083 | 1.036 | 1.000 | - | 1.072 | |  |
|  | Married | 1.000 | 1.011 | 0.994 | - | 1.029 | 1.037 | 1.008 | - | 1.065 | 1.020 | 0.998 | - | 1.044 | |  |
| **Education level** | |  |  |  |  |  |  |  |  |  |  |  |  |  | |  |
|  | Not more than high school | 1.000 | 1.019 | 0.979 | - | 1.061 | 1.024 | 0.977 | - | 1.074 | 1.048 | 0.997 | - | 1.103 | |  |
|  | University or higher | 1.000 | 1.014 | 0.997 | - | 1.030 | 1.042 | 1.016 | - | 1.069 | 1.026 | 1.002 | - | 1.050 | |  |
| **Self-report health status** | |  |  |  |  |  |  |  |  |  |  |  |  |  | |  |
|  | High | 1.000 | 1.000 | 0.989 | - | 1.012 | 1.029 | 1.004 | - | 1.054 | 1.004 | 0.988 | - | 1.020 | |  |
|  | Middle | 1.000 | 1.045 | 1.004 | - | 1.087 | 1.071 | 1.021 | - | 1.125 | 1.063 | 1.020 | - | 1.109 | |  |
|  | Low | 1.000 | 1.027 | 0.904 | - | 1.166 | 0.988 | 0.922 | - | 1.059 | 0.994 | 0.887 | - | 1.113 | |  |
| **Smoking status** | |  |  |  |  |  |  |  |  |  |  |  |  |  | |  |
|  | Non-smoker | 1.000 | 1.015 | 0.999 | - | 1.032 | 1.041 | 1.018 | - | 1.064 | 1.025 | 1.004 | - | 1.047 | |  |
|  | Ever-smoker | 1.000 | 1.027 | 0.877 | - | 1.204 | 0.972 | 0.857 | - | 1.104 | 1.022 | 0.886 | - | 1.179 | |  |
| **Sleep duration** | |  |  |  |  |  |  |  |  |  |  |  |  |  | |  |
|  | More than 7-hour | 1.000 | 1.017 | 0.997 | - | 1.037 | 1.055 | 1.025 | - | 1.086 | 1.039 | 1.013 | - | 1.067 | |  |
|  | Less than 7-hour | 1.000 | 1.016 | 0.984 | - | 1.049 | 1.000 | 0.973 | - | 1.028 | 0.999 | 0.964 | - | 1.035 | |  |
| **Physical activity** | |  |  |  |  |  |  |  |  |  |  |  |  |  | |  |
|  | No | 1.000 | 1.001 | 0.987 | - | 1.015 | 1.048 | 1.019 | - | 1.077 | 1.000 | 0.980 | - | 1.021 | |  |
|  | Yes | 1.000 | 1.039 | 1.000 | - | 1.079 | 1.031 | 0.993 | - | 1.069 | 1.047 | 1.011 | - | 1.085 | |  |

Abbreviations: * Statistically significant; aRR, adjusted Relative Risk; CI, Confidence Interval
